# UroPathogenic *Escherichia coli* (UPEC) Infections: Virulence Factors, Bladder Responses, Antibiotic, and Non-antibiotic Antimicrobial Strategies

**DOI:** 10.3389/fmicb.2017.01566

**Published:** 2017-08-15

**Authors:** Maria E. Terlizzi, Giorgio Gribaudo, Massimo E. Maffei

**Affiliations:** Department of Life Sciences and Systems Biology, University of Turin Torino, Italy

**Keywords:** urinary tract infections, uropathogenic *Escherichia coli*, bladder, antibiotics, non-antibiotic remedies

## Abstract

Urinary tract infections (UTIs) are one of the most common pathological conditions in both community and hospital settings. It has been estimated that about 150 million people worldwide develop UTI each year, with high social costs in terms of hospitalizations and medical expenses. Among the common uropathogens associated to UTIs development, UroPathogenic *Escherichia coli* (UPEC) is the primary cause. UPEC strains possess a plethora of both structural (as fimbriae, pili, curli, flagella) and secreted (toxins, iron-acquisition systems) virulence factors that contribute to their capacity to cause disease, although the ability to adhere to host epithelial cells in the urinary tract represents the most important determinant of pathogenicity. On the opposite side, the bladder epithelium shows a multifaceted array of host defenses including the urine flow and the secretion of antimicrobial substances, which represent useful tools to counteract bacterial infections. The fascinating and intricate dynamics between these players determine a complex interaction system that needs to be revealed. This review will focus on the most relevant components of UPEC arsenal of pathogenicity together with the major host responses to infection, the current approved treatment and the emergence of resistant UPEC strains, the vaccine strategies, the natural antimicrobial compounds along with innovative anti-adhesive and prophylactic approaches to prevent UTIs.

## Urinary tract infections (UTIs)

**Figure 1:**
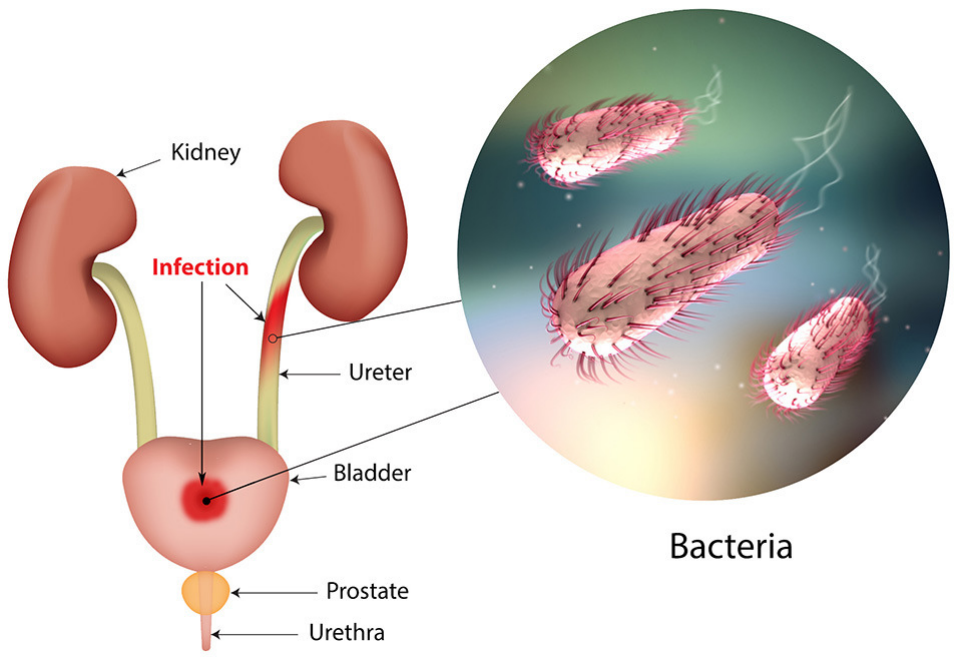
The urinary tract and sites of infection.

Urinary tract infections (UTIs) are widespread and affect a large proportion of the human population. About 150 million people worldwide develop UTI each year, with high social costs (Flores-Mireles et al., 2015). It is estimated that 40% of women develop at least one UTI during their lifetime (Micali et al., 2014) and that 11% of women over 18 years have an episode of UTI per year (Foxman and Brown, 2003; Foxman, 2014). With roughly eleven-million cases reported in the sole U.S. each year, the costs are estimated $5 billion annually (Figure 1) (Foxman, 2014).

The UTI refers to the presence of a certain number of bacteria in the urine (generally > 10^5^/ml) and symptomatic UTIs are classified in order of severity as urosepsis syndrome, pyelonephritis (or upper UTI, with infection in the kidney) and cystitis (or lower UTI, with bacteria into the bladder; Foxman, 2014; Smelov et al., 2016). Clinically, UTIs classification comprises either uncomplicated or complicated cases, depending on the presence of structural or neurological urinary tract abnormalities (Zacché and Giarenis, 2016). The ORENUC system classifies the risk factors according to the phenotype (Johansen et al., 2011): *O*, no known risk factors; *R*, risk of recurrent UTIs without a more severe outcome; *E*, extraurogenital risk factors; *N*, relevant nephropathic diseases; *U*, urologic resolvable (transient) risk factors; *C*, permanent external urinary catheter and unresolved urologic risk factors (see also Smelov et al., 2016 for a modified classification). The susceptibility to develop an UTI phenotype is related to several factors, as dysfunctions of the urinary tract and/or genetic mechanisms involved in the innate immune response control to infections (Koves and Wullt, 2016). In particular, the innate immune system may respond either to UPEC patterns (pathogen-associated molecular patterns; PAMPs) or to molecules derived from damaged or dying cells (danger/damage-associated molecular patterns; DAMPs). Pattern recognition receptors (PRRs) recognized these patterns in specialized immune cells, epithelia, and other tissues (Purves and Hughes, 2016). Assembling in the cytosol of multimeric protein complexes (inflammasomes) occurs after sensing PAMPs or DAMPs structures that can be formed in both upper and lower urinary tract (Guo et al., 2015). They trigger innate immune responses through mechanisms depending or not from the production of proinflammatory cytokines (Purves and Hughes, 2016).

The bacterial cystitis (also called acute cystitis) can occur in both women and men and some people develop recurrent infections of the urinary tract (Fiore and Fox, 2014). Three or more urinary tract infections within 12 months define the recurring UTI, as well as two or more recurrences within 6 months. The same bacterial species that caused previous infection is typically responsible for relapses. Approximately 20–30% of adult women with an initial UTI will experience a recurrence within 3–4 months; whereas, in children, about one third experiencing a UTI before the age of one, will experience a recurrence within 3 years, and 18% of them will have a recurrence within a few months (Nuutinen and Uhari, 2001). However, these figures are understated; in fact, about 50% of UTI does not come to medical attention. Recurrent UTIs can be introduced from different sources and the same or different UTI-causing strains in the gut are able to (re)inoculate the bladder. Alternatively, bacteria residing in the bladder epithelium are able to re-emerge periodically and cause UTI recurrence (Silverman et al., 2013). In patients suffering from recurrent UTIs, maintenance is ensured by antibiotic prophylaxis; however, in some cases UTI needs to be treated by surgery (Tolg and Bagli, 2012). During pregnancy, recurrent UTIs may be frequent and can cause severe adverse outcomes for the mother and the baby, including preterm birth. The interventions in this setting can be pharmacologic (antibiotics) or non-pharmacological (alternative remedies; Schneeberger et al., 2012). In pre-menopausal women, sexual activities three or more times a week, the use of spermicides, new or multiple sexual partners and having suffered from UTI before age 15 are the main risk factors in UTI development and recurrence. In menopausal women, systemic hormonal therapy is not an effective prevention and usually asymptomatic bacteriuria during this period does not require treatment (Milart et al., 2013). In women after menopause, the risk increases mainly by low estrogen levels after-effects, which are often associated to vaginal atrophy (Arnold et al., 2016). In women over the age of 61–65 years, half have suffered of genital-urinary symptoms while 29% had episodes of urinary incontinence, all symptoms associated with bacteriuria (Raz, 2001).

## Uropathogenic *Escherichia coli* and its virulence

UPEC is the main cause of community-acquired UTIs (about 80–90%; Foxman, 2014; Flores-Mireles et al., 2015). Four main UPEC phylogroups (A, B1, B2, and D) have been identified on the basis of the occurrence of genomic Pathogenicity Islands (PAI) and the expression of virulence factors, such as adhesins, toxins, surface polysaccharides, flagella, and iron-acquisition systems (Bien et al., 2012). Usually, many of these virulence factors are required for UPEC to cause UTI (Hannan et al., 2012). However, besides UPEC, UTI can be caused by *Klebsiella pneumoniae* (about 7%), *Proteus mirabilis* (about 5%), and *Pseudomonas aeruginosa, Enterococcus faecalis, Enterobacter cloacae, Streptococcus bovis*, and the fungus *Candida albicans* (for the remaining percentage; Parish and Holliday, 2012; Palou et al., 2013; Hof, 2017). During UTIs, UPEC pathogenesis includes: (a) UPEC colonization of the periurethral and vaginal areas with colonization of the urethra; (b) ascending into the bladder lumen and growth as plantktonic cells in urine; (c) adherence to the surface and interaction with the bladder epithelium defense system (see below); (d) biofilm formation; (e) invasion and replication by forming bladder Intracellular Bacterial Communities (IBCs) where quiescent intracellular reservoirs (QIRs) form and reside in the underlying urothelium; (f) kidney colonization and host tissue damage with increased risk for bacteremia/septicemia.

Replication of bacteria in the IBC can easily reach as many as 10^5^ bacteria per cell; furthermore, bacteria in the IBC undergo morphological changes, flux out of the infected cell, and go onto infect neighboring cells (Dhakal et al., 2008; Flores-Mireles et al., 2015; Spaulding and Hultgren, 2016). The flushing of urine removes most of the invading bacteria, along with UPEC-filled exfoliated bladder epithelium cells (BECs; Kaper et al., 2004).

**Figure 2:**
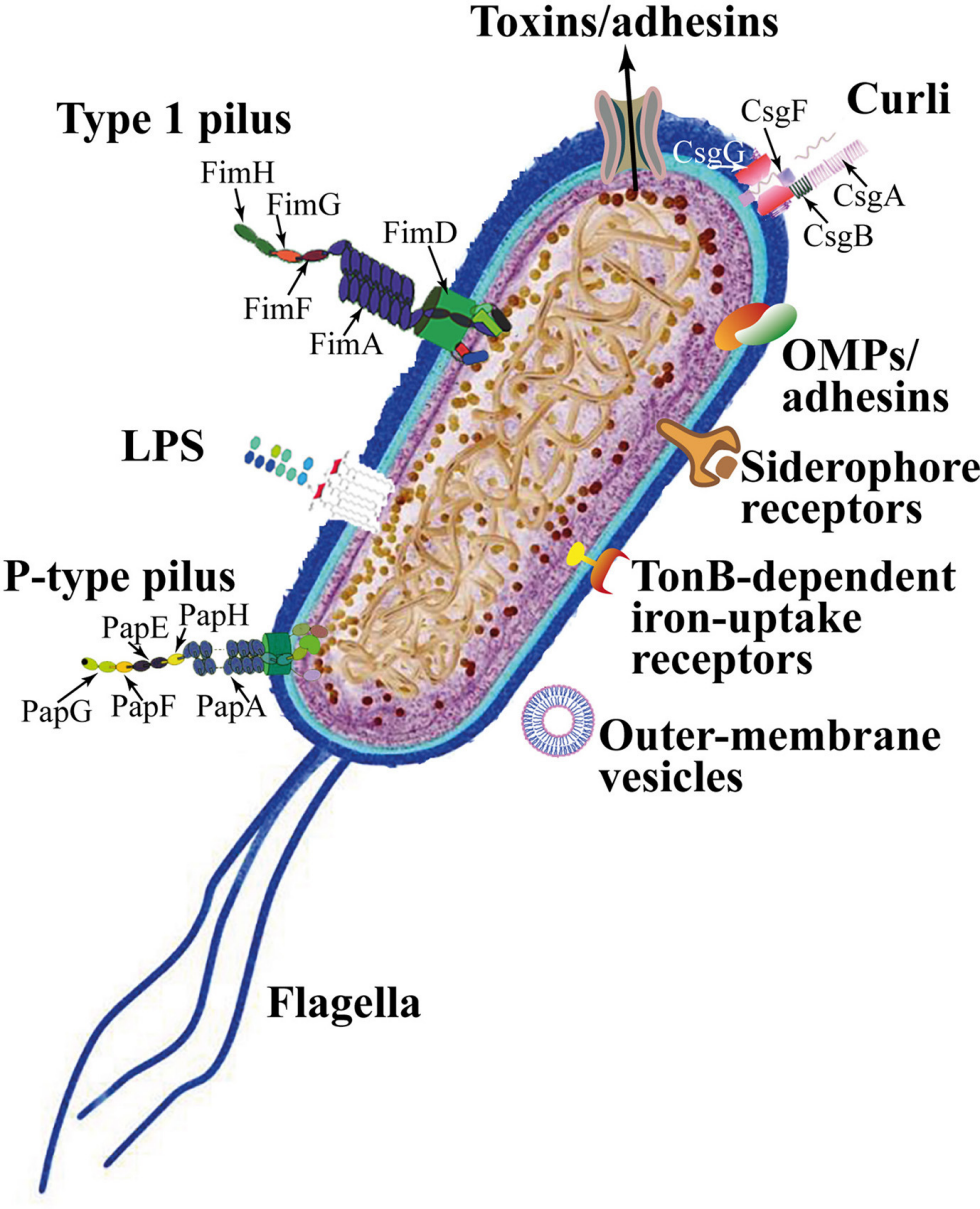
*Escherichia coli* adhesins and harboring/motile structures.

UPEC colonize the bladder using a variety of virulence factors that therefore play critical roles in UTI pathogenesis. These include surface structural components, such as lipopolysaccharide (LPS), polysaccharide capsule, flagella, outer-membrane vesicles, pili, curli, non-pilus adhesins, outer-membrane proteins (OMPs), as well as secreted toxins, secretion systems, and TonB-dependent iron-uptake receptors, including siderophore receptors (Figure 2). All of these components are attractive candidates for the development of new drugs and vaccines (Klemm et al., 2010; Werneburg et al., 2015; O'Brien et al., 2016).

LPS are molecules with amphipathic properties consisting of fatty acids lined to an oligosaccharide core, which in turn is bound to a long polysaccharide chain commonly called O antigen (Simpson et al., 2015). LPS structural constituents mediate multiple aspects of the UPEC life cycle, including the ability to acutely colonize bladders, form reservoirs, and evoke innate and adaptive immune responses (Aguiniga et al., 2016). LPS provide resistance against hydrophobic antibiotics and hypersensitivity to hydrophobic toxic molecules (such as bile salts and some antibiotics) occurs when the amount of LPS at the cell surface is decreased (Zhang et al., 2013).

In UPEC, the *fim* operon encodes type 1 pili (expressing an hemagglutination which is mannose-sensitive), whereas the *pap* operon encodes P- or Pap-pili (which are able to interact with the digalactoside unit in the P-blood group antigen). In UPEC clinical isolates, *fim* operon is constitutive whereas *pap* is part of a PAI that is also responsible for other putative virulence determinants. Generally, both types of pili are heteropolymeric consisting of a major pilus protein subunit that provides the pilus stalk and several minor subunit proteins at the distal end, with PapG and FimH representing the actual adhesins. PapG and FimH are composed by two domains, the first allows copolymerization and is made by a pilin domain, whereas the second is a lectin domain able to bind carbohydrates (Kline et al., 2009). The chaperone-usher (CU) pathway assembles pili. More than 1,000 copies of the FimA major pilin form the type 1–pilus rod, while at its distal end the pilus tip contains the FimH adhesin followed by single copies of the FimG and FimF adaptor subunits. Mannosylated proteins that are present on the bladder epithelium bind to FimH in a Rho GTPases (Rac1)-mediated host actin cytoskeleton rearrangement-dependent manner (Eto et al., 2007). This eventually leads to the development of cystitis due to bacterial invasion (Figure 2; Hahn et al., 2002). In addition, the expression of type 1 pili is strictly controlled by *phase variation*, which reversibly switches between the type 1 pili active expression (Phase-ON, piliated cells) and loss of expression (Phase-OFF, non-piliated cells; Schwan, 2011). Molecular pathways, which are involved in reversible switching between ON-OFF Phases, are strictly regulated by environmental signals within the urinary tract such as acidic pH and salt growth conditions.

Six different subunits which are arranged into two distinct subassemblies (the tip fibrillum and the pilus rod) form the P pilus. At the distal end, the tip fibrillum is composed of one PapG adhesin followed by PapF and PapE subunits. The pilus rod is made by more than 1,000 copies of the PapA subunit. The adaptor subunit PapK connects the above subunits to the PapA rod, which is a superhelical structure at the base of the pilum (Figure 2; Busch and Waksman, 2012).

Curli are bacterial surface appendages that secrete subunits from the cell as soluble monomeric proteins and possess the typical structure and physical characteristics of amyloid fibrils. which are known to be formed in some human degenerative diseases. The bacterial amyloids may facilitate biofilm formation (Goyal et al., 2014). In UPEC, curli formation is coordinated by proteins encoded in the operons *csg* DEFG. The operon-accessory proteins CsgE, CsgF, and CsgG are required to facilitate the secretion of CsgA whereas CsgB nucleates CsgA subunits into curli fibers (Figure 2; Chapman et al., 2002; Barnhart and Chapman, 2006).

While pili are involved in the initial attachment of UPEC to the urinary tract mucosa, UPEC elaborate numerous other afimbrial ahesins. In fact, the adhesin TosA is present in about 30% of urinary tract isolates and is expressed during UTI (Vigil et al., 2011). Another adhesin, FdeC, is involved in colonization of the bladder and kidneys in a mouse model of infection (Nesta et al., 2012), whereas the iron-regulated adhesin Iha mediates adherence to BECs (Johnson et al., 2005).

Moreover, the large majority of UPEC isolated from women with acute, asymptomatic, or recurrent UTIs shows the presence of flagellum-mediated motility (Wright et al., 2005). Flagella (Figure 2) are organelles that confer adhesive and invasive properties to some EPEC strains (Giron et al., 2002) and play a key role in the dynamic of biofilms (Pratt and Kolter, 1998). It was recently reported that during biofilm formation, flagella play different roles such as adherence, maturation, and dispersal as shown by gene expression and regulation during the growth phase (Nakamura et al., 2016).

On the other hand, UPEC toxins play different pathogenetic roles during infection. The α-hemolysin is in fact associated with renal damage and scarring, induces Ca^2+^ oscillations in renal tubular epithelial cells, thereby potentially enhancing ascension and colonization of ureters and kidney parenchyma by disrupting the normal flow of urine. Recently (Nagamatsu et al., 2015), α-hemolysin was found to induce proinflammatory Caspase-1/Caspase-4-dependent cell death in bladder epithelial cells, resulting in cell exfoliation (see below).

UPEC toxins, adhesins, enzymes, and non-protein antigens like LPS are not released as soluble molecules; rather, they are associated with outer-membrane vesicles, which bud off the surface of Gram-negative bacteria during all stages of growth (Figure 2; Ellis and Kuehn, 2010). The formation of membrane vesicles is considered a “smart” way to protect bacterial toxins and an efficient system to deliver them into host cell (Wiles et al., 2008).

Iron acquisition is a critical requirement for UPEC survival in an environment that is iron-limited as the urinary tract (Skaar, 2010). Thus, is not suprising that IBC UPEC show upregulation of redundant systems for the acquisition of iron (Reigstad et al., 2007). In this regard, siderophores are small-molecule iron chelators that are produced by UPEC strains to scavenge ferric iron (Fe^3+^), thus UPEC express yersiniabactin, salmochelin, and aerobactin. Siderophore receptors require the TonB cytoplasmic membrane-localized complex, a high-affinity iron acquisition system that allows binding and chelation of iron at the cell surface to promote its uptake (O'Brien et al., 2016).

However, uroepithelial cells, to prevent bacterial iron scavenging, upregulate genes for the transferrin receptor and for lipocalin 2.

Lastly, further UPEC factors associated with colonization have been linked to the regulation of metabolic pathways mediated by two-component signaling systems (TCSs). TCSs are main signal transduction pathways by which bacteria sense and respond to a wide array of environmental stimuli, including quorum sensing signals, nutrients, antibiotics. TCSs are composed by a membrane-bound sensor histidine kinase (HK) and a cytoplasmic response regulator (RR) that functions by regulating gene expression (Stock et al., 2000). Among UPEC-associated TCSs involved in UTI pathogenesis, the BarA/UvrY system has been described to regulate switching between glycolytic and gluconeogenic pathways (Tomenius et al., 2006) the EvgS/EvgA and PhoQ/PhoP systems have been involved in acid resistance (Eguchi et al., 2011), while the function of KguS/KguR is in the control of the utilization of α-ketoglutarate. In this way they facilate the adaptation of UPEC in the urinary tract (Cai et al., 2013).

The importance of the above described UPEC virulence factors in UTI pathogenesis has been further supported, in recent years, by the application of multiple “omics” technologies aimed at investigating the UPEC genomic diversity, the global gene expression in different models of infection both *in vitro* and *in vivo*, and to define the occurrence of UPEC-specific proteins as new candidate therapeutic and vaccine targets (as recently reviewed by Lo et al., 2017).

Next-generation sequencing (NGS) technologies are providing rapid low-cost determination of UPEC genomes useful to monitor outbreaks, epidemiology of emerging strains, as well as evolution of resistance (Petty et al., 2014; Stoesser et al., 2016). On the other hand, analysis of different UPEC genomes and the comparison with the *E. coli* genomic database revealed the plasticity of UPEC pan genome, and the presence of UPEC-specific PAIs genes predicted to encode putative virulence factors, such as pilus proteins, adhesins, and iron-uptake systems (Moriel et al., 2016).

Transcriptomics investigations by both microarrays and NGS-based RNA sequencing (RNA-seq), on the other hand, has led to the identification of virulence and fitness UPEC genes, expressed during different *in vitro* and *in vivo* infection-relevant conditions. In this regard, RNA-seq-based transcriptome analysis of mouse macrophages infected *in vitro* with two UPEC strains, allowed to identify strain-specific differentially expressed genes associated to the survival in macrophages, such as those involved in the responses to oxidative stress, as well as those involved in the initial adhesion of UPEC to cells, such as multiple flagella genes (Mavromatis et al., 2015). Moreover, the global gene expression of different UPEC strains has been investigated by RNA-seq of urine samples collected from UTI patients. These transcriptomics studies defined the global transcription profile for UPEC during UTI, highlighted the high genomic diversity of different UPEC strains, and confirmed, on a global scale, the expression during UTI of several genes encoding virulence factors. In fact, it has been observed the transcription of genes associated with the UPEC's adhesion to the uroepithelium (type 1 and P pili), of genes involved in iron uptake (enterobactin, aerobactin, yersiniabactin, and salmochelin), of genes encoding toxins (hemolysins nad cytotoxic factors), as well as those involved in copper efflux (Bielecki et al., 2014; Subashchandrabose et al., 2014).

High-resolution liquid chromatograph-mass spectrometry/mass spectrometry (LC-MS/MS)-based technology has been applied to identify and characterize the surface proteome of UPEC isolates and of strains grown in human urine (Wurpel et al., 2015, 2016). These studies identified several expressed proteins highly conserved among different strains, thus representing the core surface proteome of UPEC. UPEC core surface proteins, such as integral Outer Membrane (OM) proteins (e.g., OmpA, OmpC, OmpF) and several iron-uptake proteins, were in fact detected in more than 80% of strains (Wurpel et al., 2015). Clearly, characterization of those UPEC surface proteins that are conserved among different strains and immunogenic is an essential step for identifying potential vaccine candidates and new therapeutic targets (Cash, 2014).

Moreover, new insights into spatial changes in the UPEC proteome under experimental conditions mimicking bacterial growth in the urinary tract, have been provided by MALDI TOF IMS-based proteome profiling of differentially expressed proteins within UPEC biofilms. The application of this technique, that allows for in situ two-dimensional assessment of protein spatial distribution and abundance, revealed the occurrence of different bacterial subpopulations within biofilms: a type-1 pili-expressing cells localized at the air-exposed region and a curli-equipped population localized to the underlying air-liquid interface (Floyd et al., 2015).

Together, all the above mentioned “omics” approaches have allowed a great deal of new information to be available and that is enabling a more comprehensive understanding of UPEC's pathogenic mechanisms.

## The bladder epithelium shows self-defense mechanisms against invading bacteria

The most commonly targeted site of UTIs is the bladder. The bladder epithelium possesses powerful barriers and the BECs show antibacterial activities. Despite their properties, BECs and the bladder epithelium are often circumvented by UPEC (Wu et al., 2017). As discussed, the progressive ascending colonization of bacteria contaminates the urethra and the origin of this infection is usually from the gut (Kaper et al., 2004). Owing to the presence of urine, that represents an ideal growth broth, bacteria proliferate in a relatively short time lapse, while the flushing of urine during urination removes most of the invading bacteria. However, bacterial strains are able of binding tightly to BECs lining the bladder using fimbrial organelles (Duncan et al., 2004; Chahales and Thanassi, 2015).

**Figure 3:**
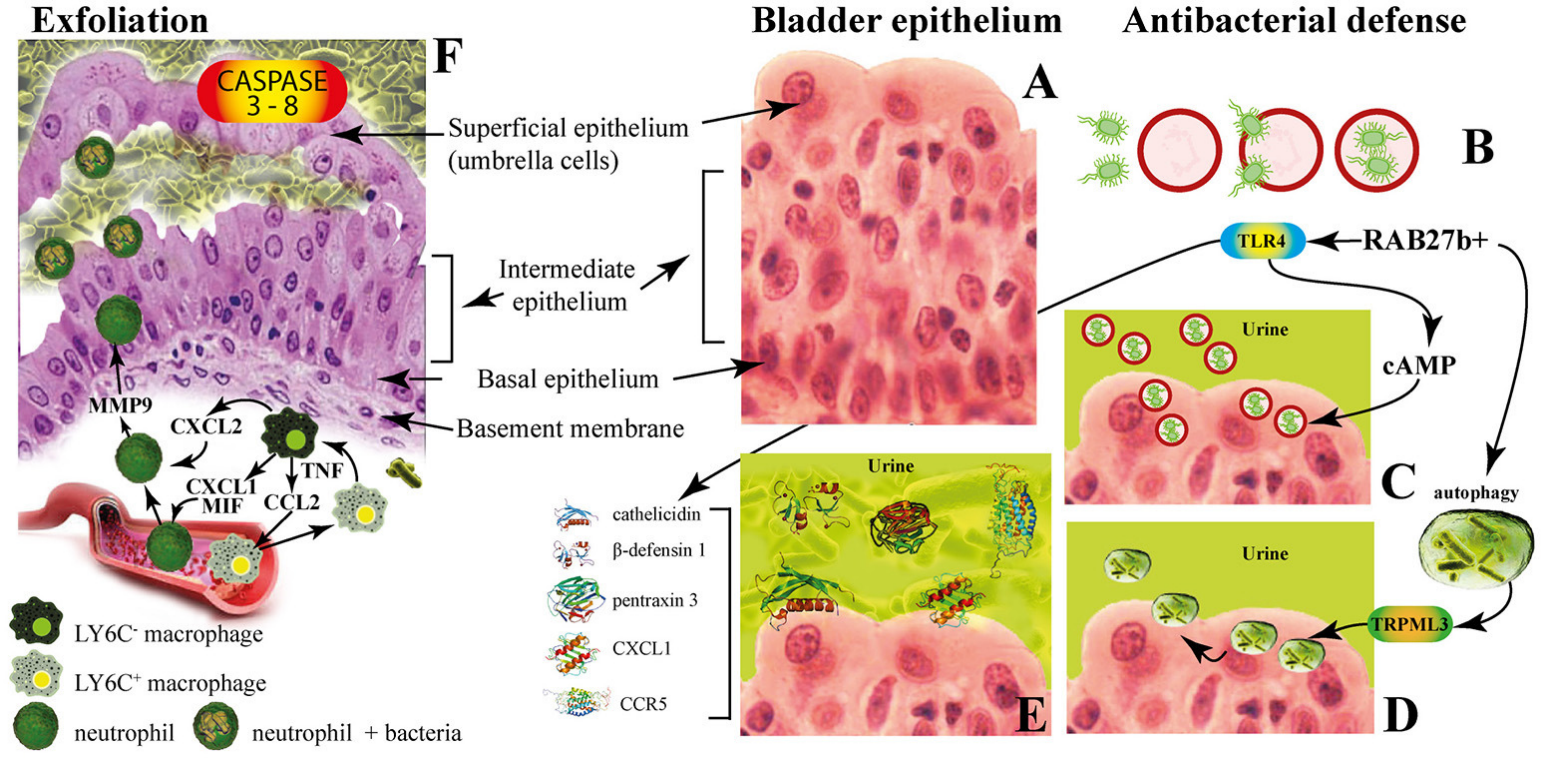
The innate immune responses of bladder epithelium to bacterial infections. **(A)** The bladder epithelium; **(B)** adherent bacteria are internalized along with Rab27b+ fusiform vesicles; **(C)** exocytosis of RAB27b+ vesicles harboring UPEC and expulsion of the intracellular UPEC back into the lumen of the bladder; **(D)** transient receptor potential mucolipin 3 Ca2+ channel (TRPML3) triggers the spontaneous expulsion of the defective lysosomes and its contents out into the extracellular space; **(E)** soluble factors are also secreted by BECs, including antimicrobial peptides (AMP, such as cathelicidin and β-defensin 1), antimicrobial proteins [such as pentraxin 3 (PTX3)] and chemokines [such as CXC-chemokine ligand 1 (CXCL1) and CC-chemokine ligand 5 (CCR5)]. **(F)** Exfoliation is accompanied by rapid renewal of superficial BECs through active proliferation of basal progenitor mast cells. Intimate crosstalk between macrophages ensures the precise initiation of neutrophil responses.

The multilayered bladder epithelium is also known as “transitional epithelium” and it is composed by three layers: basal cell layer (5–10 μm in diameter), intermediate cell layer (20 μm in diameter), and superficial apical layer with large hexagonal cells (diameters of 25–250 μm), which are also termed “umbrella cells.” A basement membrane lies underneath the basal epithelium (Figures 3A,F). The umbrella cells play a prominent role in maintaining a barrier against most substances found in urine, and show a number of properties, including specialized membrane lipids, asymmetric unit membrane particles, and a plasmalemma with stiff plaques. These plaques may cover up to 90% of the urothelial cell surface, with each plaque being composed of nearly 1,000 subunits. These subunits are made by proteins (uroplakins, UPs), which serve as the major receptors for UPEC adherence to the host cell and are localized within plaques on the apical membranes of the mature umbrella cells (Veranic et al., 2004). There is a correlation between the glycosylation changes in UPs and the different pathological conditions of the urothelium such UTI and interstitial cystitis (Birder, 2005; Katnik-Prastowska et al., 2014; Habuka et al., 2015).

The fusiform vesicles (FVs) are unique cytoplasmic organelles contained in the umbrella cells. FVs deliver preassembled crystalline arrays of UP proteins to the apical cell surface of urothelial umbrella cells. Different Rab GTPases function as regulators of specific steps in membrane traffic pathways and are localized to the cytosolic face of specific intracellular membranes. Rab27b, is a small GTPase regulating intracellular vesicle movement which is expressed at an extraordinary high level (0.1% of total protein) in urothelium. The Rab27b+ FVs are involved in the storage of extra membrane which are necessary when urine accumulates and causes bladder expansion (Wankel et al., 2016). In order to enter epithelial cells, UPEC coopt the superficial epithelial cells by expoiting their bladder volume-regulating properties by stimulating the exocytosis of fusiform vesicles right where the bacterial attach. The adherent bacteria are then internalized when these membranes are subsequently retracted into cells (Figure 3B; Wu et al., 2017). UPEC have been found to reside within Rab27b/CD63/Caveolin-1-positive fusiform vesicles (O'Brien et al., 2016). Internalized UPEC become encased in Rab27b+ fusiform vesicles within the cytosol of the superficial epithelium (Figure 3B; Bishop et al., 2007). Replication of internalized UPEC bacteria rapidly occurs, resulting in the maturation of IBCs, a structure that possesses biofilm-like properties which is protected from innate defenses and antibiotics (Justice et al., 2006; Goller and Seed, 2010). Fusion with lysosomes is thus impaired, because internalized bacteria are mostly encased in Rab27b+ compartments.

Defense mechanisms of bladder epithelial cells against intrusion of bacterial include receptors such as toll-like receptors (e.g., TLR2, TLR4, TLR5, and TLR11) that are able to promptly recognize intruding bacteria (Larue et al., 2013). After UPEC encapsulation within RAB27b+ vesicles in BECs, intracellular UPEC are recognized by TLR4 which increases intracellular cyclic AMP (cAMP) levels (Figure 3B). This triggers the exocytosis of RAB27b+ vesicles harboring UPEC and the intracellular bacterial expulsion back into the bladder lumen (Figure 3C).

However, some UPEC break the RAB27b+ vacuole and cannot be expelled into the urine; thus, these bacteria are targeted by autophagy and delivered into the lysosomes, where they actively neutralize the pH by reducing their acidicity and degradative potential (Abraham and Miao, 2015). These malfunctioning lysosomes are sensed by a lysosomal transient receptor potential mucolipin 3 Ca^2+^ channel (TRPML3), which is localized on the membrane of lysosomes (Miao et al., 2015). The activation of this Ca^2+^channel rapidly fluxes out into the cytosol the Ca^2+^ stored in the lysosome, which induces the spontaneous expulsion into the extracellular space of the defective lysosomes and its contents (Figure 3D).

Pathogen sensing by TLR4 induces the production of various soluble factors which are secreted by BECs, including antimicrobial peptides (AMP, such as cathelicidin and β-defensin 1; Sun et al., 2013; Chromek, 2015), antimicrobial proteins [such as pentraxin 3 (PTX3); (Uzun et al., 2016)] and chemokines [such as CXC-chemokine ligand 1 (CXCL1) and CC-chemokine ligand 5 (CCR5); Schiwon et al., 2014; Figure 3E]. Attachment to the urothelium or bacterial lysis are inhibited by these antimicrobial peptides, which are also induced when bacteria succeed to attach to the urothelium (Spencer et al., 2014). Moreover, excretion in the urine of uromodulin, a major high mannose-containing glycoprotein, exerts a protective effects against UTI by competing with the binding of UPEC FimH to uroplakin Ia (Pak et al., 2001).

When all these export mechanisms fail to clear the urothelium from the invading UPEC, BECs activate the last line of defense. Acute infections are commonly associated with of the exfoliation of the epithelium, with the loss of a large numbers of superficial epithelial cells. Exfoliation is followed by an efficient restoring of superficial BECs through active proliferation of basal progenitor mast cells (MCs). BECs exposed to UPEC release copious amounts of interleukin-1β (IL-1β) that regulates migration of multiple cell types including neutrophils and MCs (Choi et al., 2016). Exfoliation is also triggered by caspase 3- and caspase 8-dependent apoptosis of infected BECs, which shed into the bladder lumen (Figure 3F). However, exfoliation, which is an efficient host defense strategy, may is some cases favor the dissemination of bacteria, by clearing the way to deeper tissues. Indeed, the death of the superficial epithelium is intentionally induced by certain virulent UPEC to better reach deeper tissue where intermediate BECs are located and where they form QIRs and where they can persist for extended period of time. Actually, one of the main reason for high rate recurrence of infections in the bladder and resistance to antibiotics is associated to the presence of QIRs within subepithelium (Leatham-Jensen et al., 2016).

The immune system operates with different and specific strategies to reduce inflammation and to preserve tissue integrity. The direct phagocytosis of bacteria is operated by neutrophils that also clear bacteria through extracellular burst of ROS, which are highly toxic to bacteria (Aubron et al., 2012). Intimate crosstalk between LY6C− and LY6C+ macrophages ensures the precise initiation of neutrophil responses (Figure 3E). Local LY6C− macrophages release CC-chemokine ligand 2 (CCL2), CXC-chemokine ligand 1 (CXCL1) and macrophage migration inhibitory factor (MIF) to recruit LY6C+ macrophages and neutrophils from the bloodstream (Schiwon et al., 2014; Figure 3F). LY6C+ macrophages, as a consequence of infection sensing, secrete tumor necrosis factor (TNF), which acts on local LY6C− macrophages to trigger their production of CXCL2. The last is responsible for spontaneously production of matrix metalloproteinase 9 (MMP9) by neutrophils and their transepithelial movement (Nathan, 2006). The resident LY6C− macrophages play a major role as the main pro-inflammatory cells, whereas the recruited LY6C+ macrophages keep neutrophils in close proximity before targeting the pathogen (Abraham and Miao, 2015).

## UPEC antibiotic susceptibility and resistance

The efficacy of antibiotic treatment depends on the identification and antimicrobial resistance pattern of uropathogens responsible for UTI (Bartoletti et al., 2016). The practice of prescribing antibiotics to treat UTI without bacterial characterization led to increased resistance among uropathogens and to decreased effectiveness of oral therapies. Despite clinical symptoms of UTIs have been ameliorated by numerous antibiotics, UPEC persistence and resistance to antibiotics represent a serious problem (Blango and Mulvey, 2010). According to the 2015 guidelines of the European Association of Urology, the recommendations for the prevention of recurrent UTI are first aimed at behavioral changes and immediately after toward non-antibiotic measures. If these two recommendations are not sufficiently effective then the antibiotic prophylaxis should be considered, in order to prevent the adverse events and collateral damages that the long-term and not necessary use of antibiotics may cause (Vahlensieck et al., 2016). In Europe, resistance to UPEC isolates shows average values of 11.8% for third-generation cephalosporins and 22.3% for fluoroquinolones. In the U.S., fluoroquinolone-resistant UPEC represented the 31.3% of isolates among hospitalized patients between the years 2007 and 2010 (Edelsberg et al., 2014). These data confirm the general consideration that number of effective antibiotic compounds availability and the prevalence of antibiotic resistance are worsening, as demonstrated by an increased number of clinical studies (Bartoletti et al., 2016).

Antimicrobial prophylaxis for women with recurrent UTI include, for example, 50 mg or 100 mg of nitrofurantoin once a day; 100 mg of Trimethoprim (TMP) once a day; 40/200 mg TMP/sulfamethoxazole (co-trimoxazole) once a day or three times a week; 3 g of fosfomycin trometamol every 10 days and, during pregnancy, for example, 125–250 mg of cephalexin or cefaclor 250 mg once a day (Grabe et al., 2015; Giancola et al., 2017). Among other antibiotics, imipenem represents the best efficient antibiotic against all UPEC strains (100%), followed by ertapenem (99.98%), amikacin (99.94%), and nitrofurantoin (99.91%). Carbapenems like imipenem represent the best option for the treatment of extended-spectrum beta-lactamase (ESBL) strains (Idil et al., 2016). UPEC strains are also susceptible to ciprofloxacin (Tosun et al., 2016), cefotaxime, piperacillin/tazobactam (Dizbay et al., 2016), azithromycin, doxycycline and ceftriaxone (Saha et al., 2015). However, several UPEC isolates are resistant to ampicillin, oral first-generation cephalosporins, TMP-sulfamethoxazole (Moya-Dionisio et al., 2016), cefuroxime (Chang et al., 2016), cotrimoxazole (Saha et al., 2015), amoxicillin-clavulanate, nalidixic acid, cefradine, and aminopenicillins (Narchi and Al-Hamdani, 2010). In some cases, the combined effect of different antibiotics prompted a significant increment in susceptibility, as found for triclosan with amoxicillin and gentamicin (Wignall et al., 2008). A retrospective analysis has identified ciprofloxacin as the most used antibiotic for empirical therapies (76% of cases; Parish and Holliday, 2012).

Due to ecological side effects, the oral cephalosporins and fluoroquinolones are no longer recommended as routine treatments, except for specific clinical situations. Furthermore, the worldwide increment of UPEC strains resistant to TMP questions its use with or without a sulfonamide as an effective prophylactic agent (Idil et al., 2016). High urinary levels of levofloxacin are not enough to cure UTIs and the combination of ceftolozane/tazobactam was more effective as an alternative treatment in settings of increased fluoroquinolone resistance (Huntington et al., 2016). Increased resistance of UPEC strain isolates against ampicillin (96.42 %), tetracycline (85.71 %), amikacin (71.42 %), ciprofloxacin (67.85 %), and gentamycin (58.71 %) has been found in pregnant women with history of recurrent UTIs (Habibi and Khameneie, 2016). Finally, numerous government agencies advise against the long-term use of prophylactic nitrofurantoin because of rare but serious pulmonary and hepatic adverse effects (Vahlensieck et al., 2016).

New antibiotics, such as colistin (Cui et al., 2016), finafloxacin, and cefiderocol (S-649266), which are currently in early clinical development, might be useful in the treatment of UTIs (Zacché and Giarenis, 2016).

**Figure 4:**
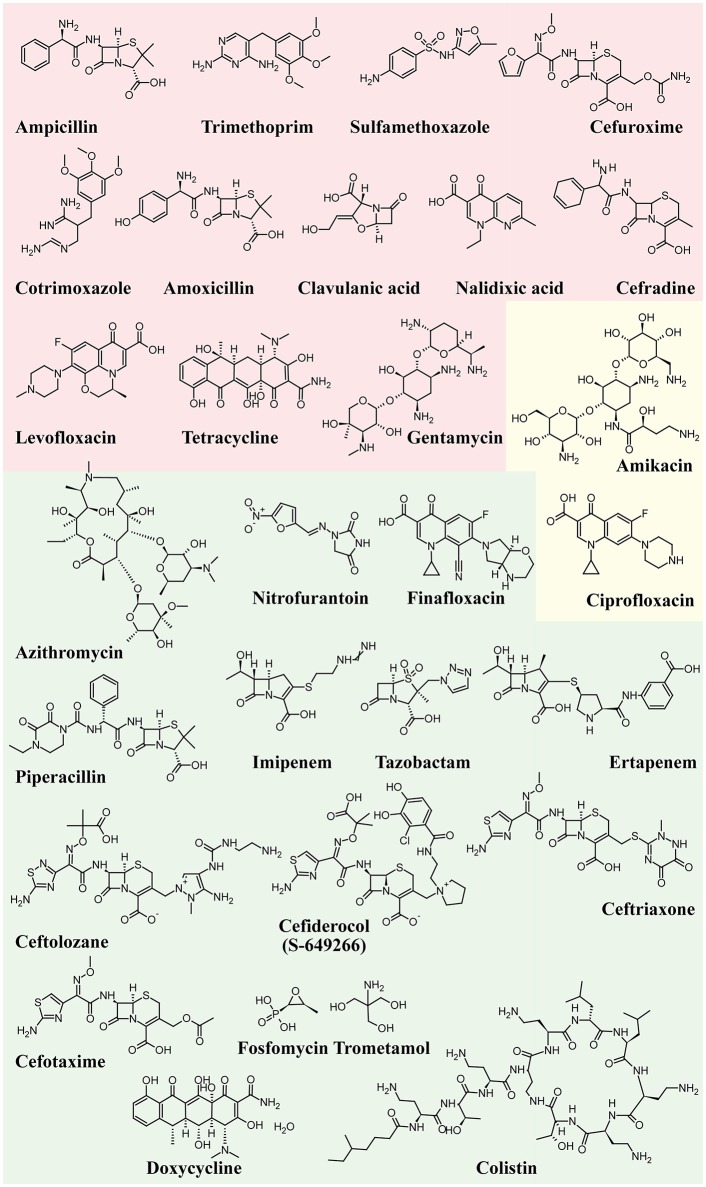
Structure formulae of some UPEC resistant and susceptible antibiotics. UPEC resistance is shown with a red background, susceptibility with a green background, whereas the yellow background shows antibiotics that already show resistance in some UPEC strains.

Figure 4 shows the structure formulae of the most representative antibiotics for which UPEC resistance has been demonstrated (red background) and those showing susceptibility to UPEC (green background). The yellow background shows antibiotics that already show resistance in some UPEC strains.

## Alternative antimicrobial remedies

Antibiotics will continue to be an unavoidable source for the prevention of UTIs on a case-by-case basis. However, the excessive use of antibiotics and the long-term interference with intestinal microbiota, require to search for alternative remedies. A plethora of molecules has been tested to reduce UPEC infections by exploiting their ability either to stimulate the immune system or to interfere with the UPEC ability to adhere and invade the urothelium. Here we briefly summarize the most effective alternative remedies to fight UPECs.

### Vaccines

The development of new strategies to fight UTI has focused on the development of vaccines based on bacterial components with the aim of identifying specific UPEC factors for potential use as vaccine antigens (McLellan and Hunstad, 2016). Among candidate antigens, potential targets are adhesins, antimicrobial peptides (AMPs), and siderophores (Spaulding and Hultgren, 2016). However, the use of vaccines may alter the proteobacteria populations of *E. coli* in the gut and may find a difficult way to reach the bladder lumen. Furthermore, vaccine use might be more efficient to treat upper rather than lower urinary tracts (McLellan and Hunstad, 2016). Recently, healthy adult women with a history of recurrent UTI where the subject of a multicentre phase 1b trial where a single intramuscular injection of either a bioconjugate vaccine containing the O-antigens of four *E. coli* serotypes (ExPEC4V) or placebo were administered. Vaccination induced significant IgG responses for all serotypes; moreover, the vaccine group showed significantly lower UTIs caused by UPEC of any serotype when compared with the placebo group (Huttner et al., 2017). In a meta-analysis of about 900 patients, the oral vaccine OM-89 (Uro-Vaxom®) reduced the mean number of UTIs by half, whereas a vaginal vaccine (Urovac) showed a scanty reduction in recurrent UTIs and caused a vaginal irritation in nearly 28% of patients (Beerepoot et al., 2013). An alternative strategy to elicit protective immunity is to select as antigens small molecules, rather than proteins or peptides. The use of siderophore-protein conjugates was found to elicit immune responses targeted to bacterial siderophores and to successfully protect against UTI (Mike et al., 2016). A reverse-vaccinology approach in combination with proteomics and genomics was used to identify putative broadly protective vaccine antigens (Moriel et al., 2016). Currently, no UTI vaccines are approved in the United States but among current strategies, immunotherapeutic formulation OM-89 (marketed in Europe by EurimPharm GmbH as UroVaxom), which is a bacterial extract prepared from 18 strains of *E. coli*, is really promising (Neto et al., 2016). The identification of potential vaccine targets has been recently reviewed (O'Brien et al., 2016; Poolman and Wacker, 2016).

### Probiotics

Women with recurrent UTIs often show alterations in their vaginal or periurethral microbiota (Czaja et al., 2009). Probiotics have been extensively used as alternative approaches to reduce recurrent UTIs (Zacché and Giarenis, 2016). Several *Lactobacilli* strains showed UPEC inhibitory activity between that of sensitive and resistant antibiotics (Shim et al., 2016). One of the major roles of *Lactobacilli* is their ability to clear potential UPEC reservoirs, thus preventing recurrences. *Lactobacillus*-mediated protection from UTI is not clear, and may involve hydrogen peroxide, surfactants, and anti-adhesive molecules production (O'Brien et al., 2016). However, contrasting results have been reported. For instance, *Lactobacilli* prophylaxis did not decrease the rate of UTI recurrence in several open randomized trials with women who had a UTI caused by UPEC (Kontiokari et al., 2001; Beerepoot et al., 2013; Schwenger et al., 2015). On the other hand, *Lactobacilli* were shown to actively stimulate the immune system by up- and down-regulation of NF-κB activity (Karlsson et al., 2012), and to colonize and protect the vagina (Reid, 2000), thus suggesting some sort of indirect-cooperative function.

### Estrogens

The vaginal epithelium and its acidic microenvironment provide substantial inhibition of bacterial growth of enteric microorganisms. Estrogen is an important modulator of urothelium cell growth and differentiation. Estrogen may constitute a risk factor for infections in young women; however, after menopause the low estradiol levels have been related to recurrent infections (Mody and Juthani-Mehta, 2014). Estrogen application modulates two epithelial defense mechanisms: induction of AMPs and reduction of epithelial exfoliation (Luthje et al., 2013). Furthermore, increased epithelial integrity and higher expression of AMPs may reduce the formation of QIRs as the source of recurrent infections (Luthje and Brauner, 2016). However, oral estrogen therapy failed to be effective at reducing UTI risk compared with placebo, whereas vaginal estrogen application reduced UTI (Perrotta et al., 2008; Matulay et al., 2016). Vaginal estrogen therapy was found to be effective in preventing recurrent UTI of postmenopausal women when used in combination with hyaluronic acid, chondroitin sulfate, curcumin, and quercetin administered per os (Torella et al., 2016).

### Pilicides and curlicides

The requirement of pili for adhesion by UPEC makes inhibitors of the assembling chaperone-usher pathway (CUP) as potential targets to reduce UTI (Aberg and Almqvist, 2007). Therefore, pilicides represent an interesting alternative to antibiotics. Two classes of pilicides have been developed: amino acid derivatives and pyridinones (Svensson et al., 2001). In laboratory and clinical *E. coli* strains, these compounds have been demonstrated to be able to reduce by almost 90% hemagglutination mediated by either type 1 or P-pili adherence to BECs and biofilm formation mediated by type I pili (Pinkner et al., 2006). One of these pilicides, ec240, was found to decrease motility and dysregulate CUP pili, including type 1, P, and S pili (Greene et al., 2014).

Curli-mediated biofilm formation requires a specific assembly machinery (Chapman et al., 2002). Curlicides are inhibitors of both type 1 pilus production and curli biogenesis. Compounds derived from the peptidomimetic scaffold that show pilicide activity can prevent both Aβ aggregation and curli formation (Chorell et al., 2011). A small-molecule curlicide (FN075) was shown to inhibit both type 1 pilus production and curli biogenesis by reducing the biofilm and virulence of UPEC in a mouse model of experimental cystitis (Cegelski et al., 2009).

**Figure 5:**
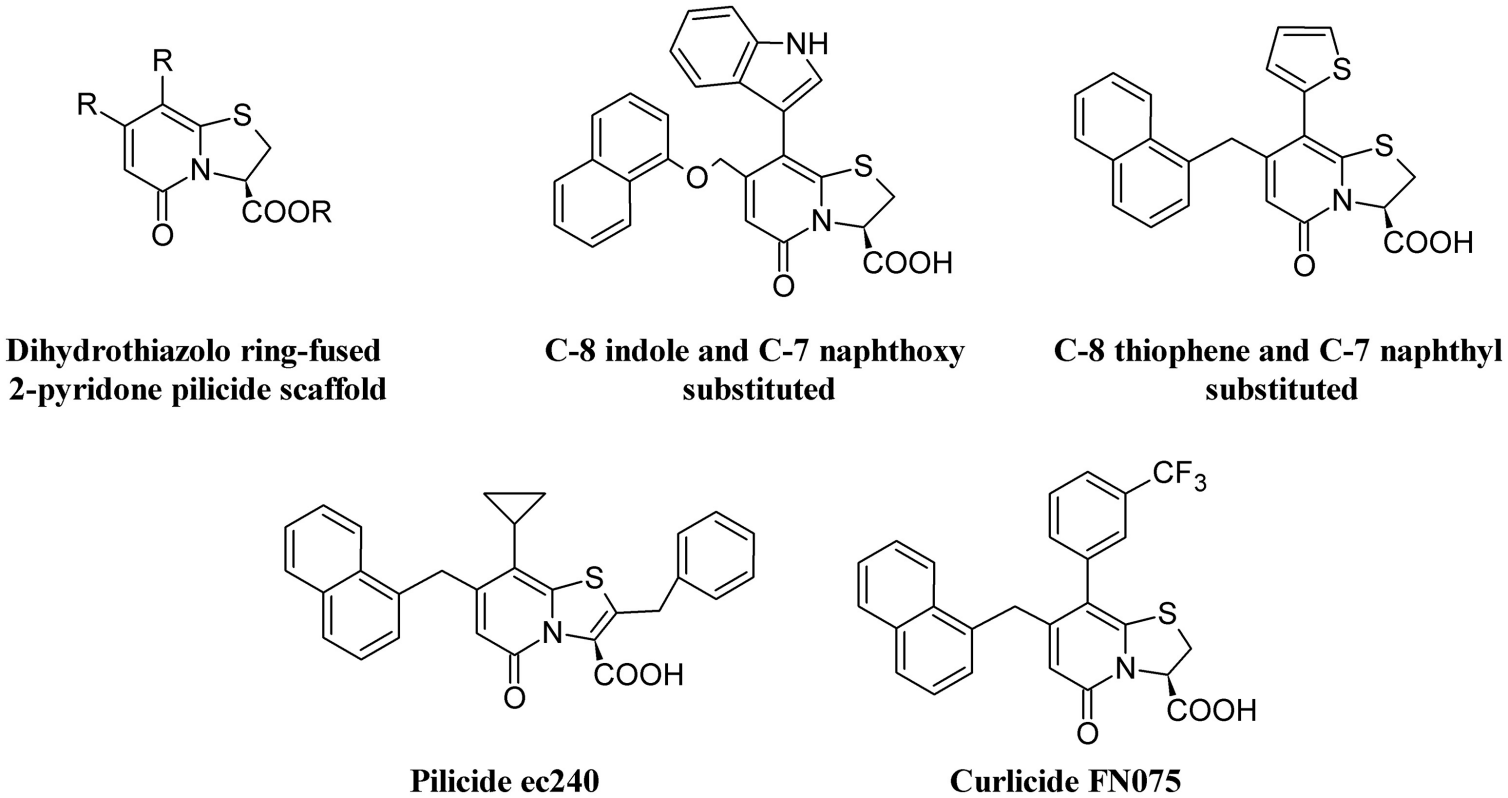
Structure formulae of pilicide scaffold, some bioactive pilicides, and the curlicide FN075.

Figure 5 depicts the chemical structure of some pilicides and curlicides.

### d-Mannose and d-Mannose-derived FimH antagonists

**Figure 6:**
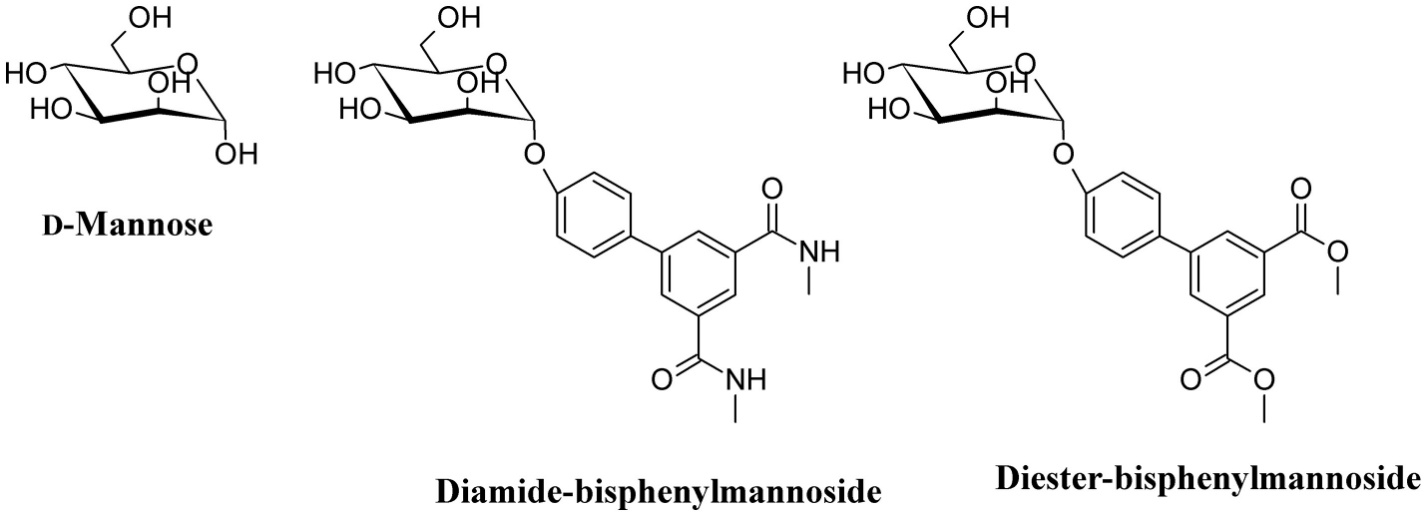
Structure formulae of d-mannose and some bioactive mannosides.

One of the main strategies to reduce UPEC infection is targeting bacterial adhesion by inhibiting, for instance, FimH. By using catch bond binding mechanisms, UPEC Type I fimbriae FimH binds terminal epitopes of high mannose and paucimannosidic glycans conjugated to uroplakin Ia which are located on the surface of urothelial cells (Sauer et al., 2016). The x-ray crystal structures of FimH bound to α-D-mannose, and mannose derivatives have been used to rationally design specific FimH inhibitors (Han et al., 2012). d-Mannose (Figure 6) is involved in the glycosylation of some proteins; this molecule is a C-2 epimer of d-glucose that play several roles in the human metabolism. Mutation in enzymes involved in the mannose metabolism induces certain glycosylation disorders (Gordon, 2000). The use of d-Mannose as a dietary supplement has the intent of influencing the glyconutrient status and improve human health (Hu et al., 2016). In both *in vivo* and *in vitro* studies, the transport rate of d-mannose across the intestine was found to be approximately one tenth that of d-glucose (Duran et al., 2004). d-mannose can bind proteins to induce macrophage activation and interleukin-l release (Hu et al., 2016), but its most important action with respect to UTI is the capability to saturate FimH adhesin by blocking the invariant lectin pocket (O'Brien et al., 2016; Zacché and Giarenis, 2016). However, side effects of d-mannose have been reported underscoring the importance of stringent regulation of d-mannose metabolism, particularly for a subset of pregnant women (Freinkel et al., 1984; Sharma et al., 2014a,b). The only published clinical study on d-mannose effect in UTIs reduction indicates similar effects of nitrofurantoin, with no significant side effects when compared to the antibiotic treatment. However, this study suffers of a low number of recruited patients (Kranjcec et al., 2014).

Mannosides are small-molecular weight molecules that are orally bioavailable and show inhibiting action toward the FimH adhesion; murine models show that these molecules are highly efficacious in the treatment of UTI (Cusumano et al., 2011; Han et al., 2012). Mannosides are a cost-effective treatment able to lower the antibiotic resistance rate and represent interesting therapeutic compounds for the treatment and prevention of UTIs (Kostakioti et al., 2012). For instance, several mannosides (Figure 6) are able to attenuate UPEC virulence through the blockage of FimH binding to BECs. This binding prevents bacterial adherence, invasion, and IBC formation (Han et al., 2010).

### Vitamin D

**Figure 7:**
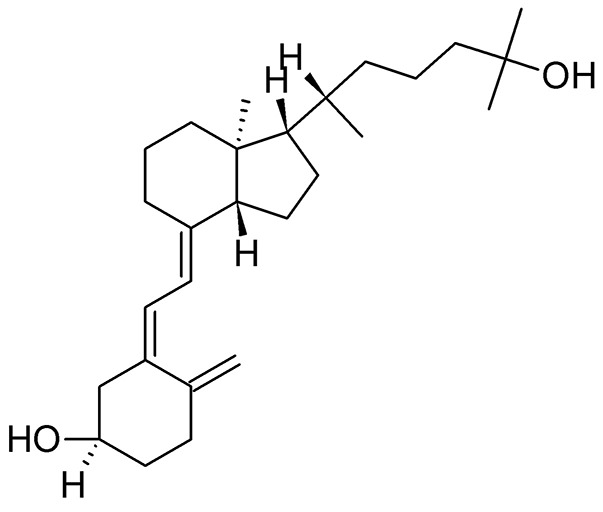
Structure formula of 25-hydroxyvitamin D_3_.

As discussed above, AMPs exhibit alternative functions and play an important supporting role in the immune system. The human antimicrobially-active AMP cathelicidin is made by 37 amino acids and is referred to as LL-37 because of the starting with two lysin residues. LL-37 binds Curli fimbriae of UPEC and prevents interaction with the bacterial cell membrane (Kai-Larsen et al., 2010). The transcription factor vitamin D receptor (VDR) is a direct target of the gene that encodes for cathelicidin antimicrobial peptide (*CAMP*) and up-regulation of *CAMP* is induced by VDR in response to 25-hydroxyvitamin D_3_ (vitamin D_3_, Figure 7) and its analogs (Gombart et al., 2005). Therefore, Vitamin D has the potential to protect the urinary tract against infection by modulating the production of AMPs. Both pediatric and premenopausal studies revealed that Vitamin D deficiency correlates with a higher frequency and severity of UTI (Nseir et al., 2013; Hacihamdioglu et al., 2016), suggesting a promising role of vitamin D as a potential complement in the prevention of UTI (Hertting et al., 2010; Luthje and Brauner, 2016).

### Methenamine

**Figure 8:**
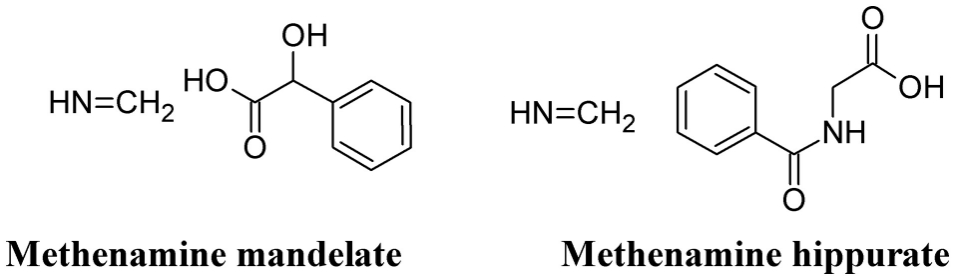
Structure formulae of methenamine mandelate and hippurate.

Methenamine, when used in combination with mandelic or hippuric acid (Figure 8) is used for the treatment of UTIs (Cronberg et al., 1987). In the urine, the presence of acidifying substances (such as organic acids and Vitamin C) lowers the pH (≤ 6.0) and decomposes methenamine to form formaldehyde and ammonia, the former exhibiting an aspecific bactericidal effect. Because of some adverse effects (mainly chemically-induced hemorrhagic cystitis in overdose), the use of this compound has been reduced. However, because of the recurrence of UTI and the increased antibiotic resistance of UPEC, methenamine has been re-introduced in substitution to the use of daily antibiotic prophylaxis. Moreover, methenamine was not found to contribute to overall increase in resistance and can be generally considered as safe and well-tolerated (Matulay et al., 2016).

### Phenols and polyphenols

Phenolic compounds exert a strong antibiotic effect and can be generally subdivided into polymeric and not polymeric phenolics.

#### Polymeric phenolics

**Figure 9:**
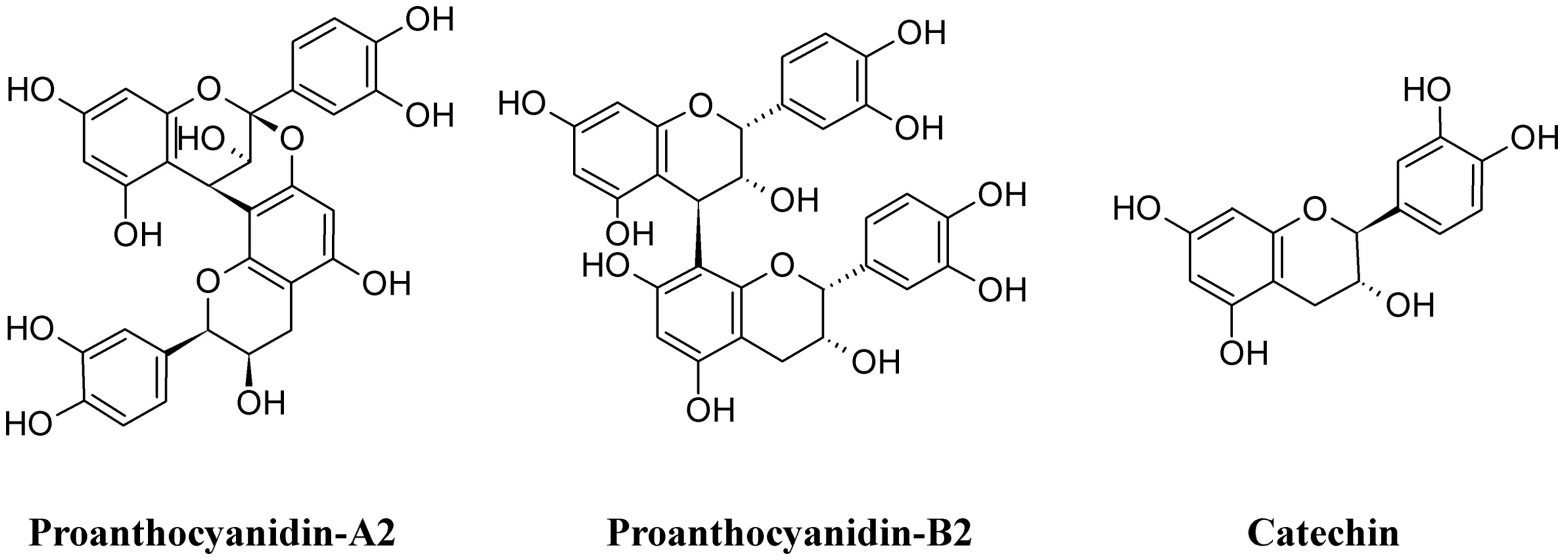
Structure formulae of some polymeric phenolics.

Among polymeric phenolics, proanthocyanidins (PACs) represent an interesting class of compounds. These tannins are produced by different plants and those isolated from cranberry (*Vaccinium macrocarpon*) are particularly rich in A-type linkages (PAC-A), compared to the more common B-type linkages of other proanthocyanidins (PAC-B; Figure 9; Chughtai et al., 2016). PACs-A were found to inhibit P-fimbrial adhesion *in vitro* and to play a significant role in UTI prevention (Howell et al., 2005; Silverman et al., 2013; Occhipinti et al., 2016). Despite the considerable number of scientific and clinical studies to support efficacy of cranberry extracts in reducing UTI, inconsistency in meta-analysis methodologies, clinical heterogeneity (i.e., participants, the result and the intervention) and methodology (i.e., the trial design and execution and inclusion/exclusion criteria) along with the poor characterization of the cranberry extract used may lead to different results and interpretations, as recently pointed out (Chrubasik-Hausmann et al., 2014; Liska et al., 2016; Nicolle, 2016). Cranberry extracts containing 72 mg PAC-A produce an active and significant bacterial anti-adhesion in human urine (Howell et al., 2010; Micali et al., 2014; Singh et al., 2016); however, the mechanism by which PACs-A exert their anti-adhesive action is not yet clear. For instance, the conserved lipid A moiety of LPS are preferentially recognized by PACs, enabling them to bind LPS from multiple Gram-negative bacterial species (Delehanty et al., 2007). Recently, a standardized cranberry extract particularly rich in PAC-A and its purified PAC-A fractions were tested for their antiviral activity against Herpes 1 and Herpes 2. By using a combination of molecular and biochemical analyses, Terlizzi and co-workers were able to show that PAC-A was able to specifically target viral glycoprotein gD and gB, thus causing the inability of viral particles to infect target cells (Terlizzi et al., 2016). These new findings open the possibility that PAC-A may not only target the FimH lectins as d-mannose. Recently, adherence of UPEC to the bladder was enhanced in the surfactant protein A (SP-A)-deficient mice; therefore, SP-A may play an important role in innate immunity against UPEC (Hashimoto et al., 2017). Because dimers and trimers of PAC-A can enter the bloodstream (Foo et al., 2000; Feliciano et al., 2012, 2014; Zhang et al., 2016) they can potentially reach urothelial cells. Owing to the ability of these polyphenols to enter the cells and actively interact (Menghini et al., 2011; Vu et al., 2012) it is conceivable to argue the possibility of a PAC-A effect on SP-A, limiting bacterial adherence. However, further studies are necessary to dissect the role of PAC-A in UTIs reduction.

Green tea extracts are also rich in polyphenols, with catechins (Figure 9) being the most active components. The antimicrobial activity of green tea against UPEC was correlated to the ability of catechins to be excreted in the urine at concentrations enough to reduce the UTI (Reygaert and Jusufi, 2013), and this hypothesis was confirmed by intravescical instillation on a rat model of bacterial cystitis (Rosenberg et al., 2014).

#### Non-polymeric phenolics

**Figure 10:**
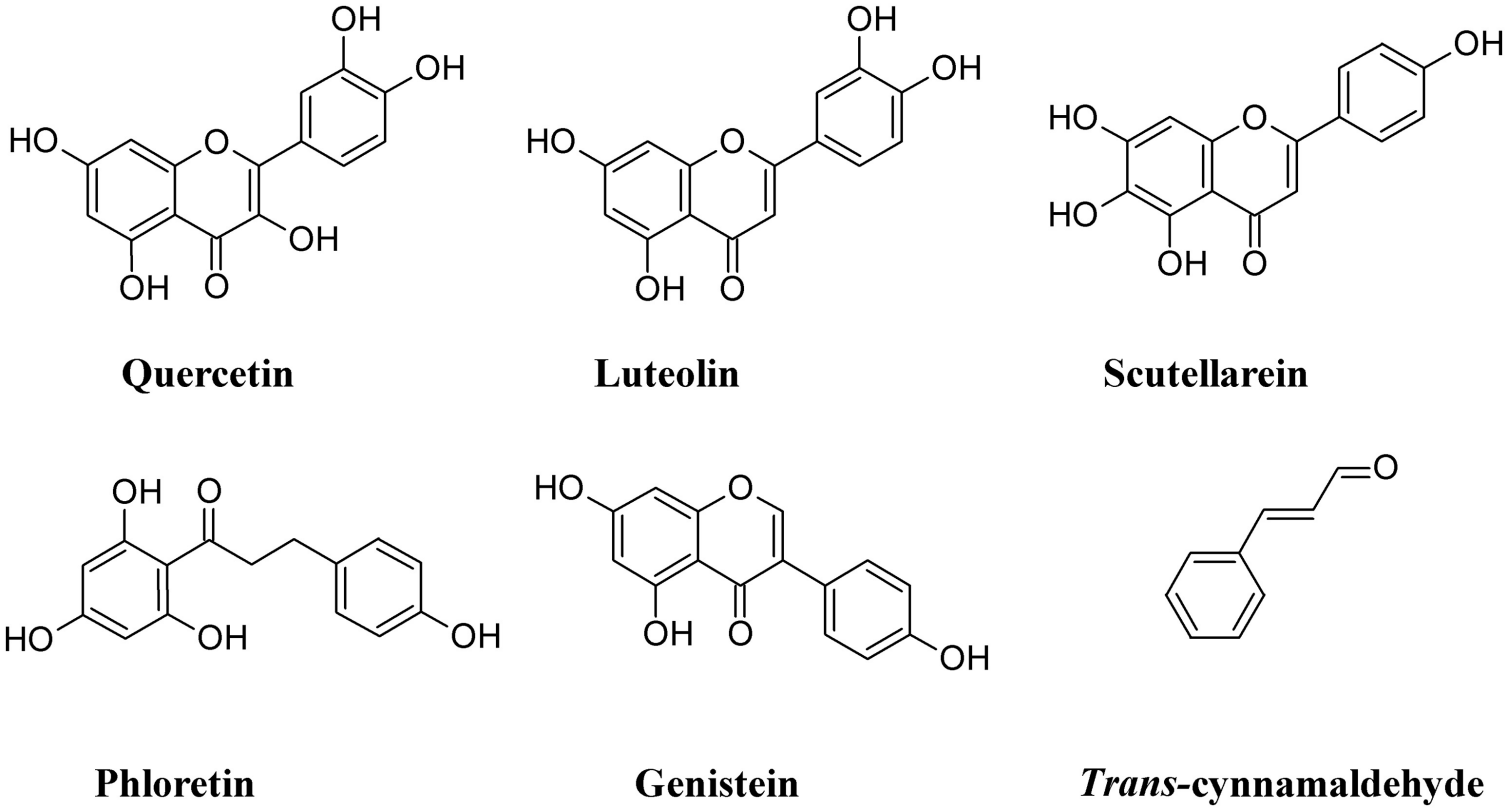
Structure formulae of some non-polymeric phenolics.

One of the cellular mechanisms that increase resistance to UPEC invasion of BECs is the inactivation of the Rac1-GTPase and the activation of the adenylyl cyclases, the latter catalyzing the conversion of ATP to cAMP (Bishop et al., 2007; Song et al., 2007). Some non-polymeric flavonoids such as quercetin, luteolin, scutellarein, phloretin, and genistein, are present in the diet and are excreted in the urinary system (Wang et al., 2016). These compounds inhibit the activity of cAMP and cyclic nucleotide phosphodiesterases (PDEs; Kuppusamy and Das, 1992) and are considered as potential non-antibiotic therapeutic agents in UTI setting. Quercetin has a wide range of biological properties and has been used to prevent interstitial cystitis (Theoharides et al., 2008). Luteolin, by interfering with PDE activity, inhibits lymphocyte function-associated antigen-1 (LFA-1) expression and neutrophil adhesion to endothelial cells, thus acting as an antinflammatory agent (Jiang et al., 2015) and a protectant of bladder epithelial cells against UPEC (Shen et al., 2016). Furthermore, this flavone inhibits UPEC-induced cytotoxicity and decreases both attachment and biofilm formation in pre-treated UPEC (Shen et al., 2014). Bacterial fatty acid synthesis, in particular β-ketoacyl acyl carrier protein synthase (KAS) enzymes, are good therapeutic targets for novel antibiotics. *In silico* studies revealed that binding energy toward KAS I active site of the flavonoinds genistein and isorhamnetin can be used as potential drug candidates against UPEC (Figure 10) (Sabbagh and Berakdar, 2015).

Other non-polymeric phenolic exert antiadhesive properties. *Trans*-cinnamaldehyde is able to down-regulate the expression of some UPEC genes involved in attachment and invasion of host tissue. This compound was found to significantly decrease UPEC attachment and BECs invasion (Amalaradjou et al., 2011).

### Other medicinal plants with antibacterial activity against UPEC

Several studies report the antibacterial activity of plant extracts, particularly in *in vitro* studies (Luthje and Brauner, 2016). In the majority of these studies, the failure to identify the main compound responsible for the reduction of UPEC adhesion or any other possible mechanism involved in alleviation of UTI makes these studies interesting from a general phytochemical viewpoint, but less useful to the understanding of mechanism of action. Table 1 summarizes the effects of different plant extracts on UTI and reduction of UPEC infection and adhesion to BECs.

**Table 1:** Plant extracts with antimicrobial/antiadhesive activity against UPEC.

**Botanical name**	**Part used**	**Compound class**	**Compound**	**Effect**	***In vivo/in vitro/*clinical**	**Mechanisms of action**	**References**
*Agropyron repens*	Rhizome	None identified	None identified	Decreased bacterial adhesion	*In vivo*	Interaction with bacterial outer membrane proteins	Rafsanjany et al., 2013
*Alchornea cordifolia*	Leaves and stem bark	Terpenoids, phenolics	Friedelane-3-one-28-al, stigmasta-4,22-dien-3-one, friedelin, 3-O-acetyl-aleuritolic acid, 3-O-acetyl-erythrodiol, methyl gallate	Antibacterial activity on ESBL-producing *E. coli* isolates	*In vitro*	None	Noundou et al., 2016
*Andrographis paniculata*	Leaves	Terpenoid	Andrographolide	Inhibits LPS-induced iNOS and COX-2 proteins expression, production of nitrite, LPS-mediated TNF-α, IL-1β and IL-6 production	*In vitro*	Negative regulation involving STAT3 phophorylation and NF-κB activation	Lee et al., 2011
*Arctostaphylos uva-ursi*	Leaves	Phenolics	Arbutin, hydroquinone conjugates	UTI control	*In vitro*	Shrinking and tightening of mucous membranes	De Arriba et al., 2013; Dietz et al., 2016
*Aristolochia indica*	Whole plant	None identified	None identified	Antibacterial Activity against MDR UPEC	*In vitro*	None	Venkatadri et al., 2015
*Armoraciae rusticanae*	Roots	Isothiocyanates	Benzyl-Isothiocyanate, Phenylethyl-Isothiocyanate	Intermediate susceptibility,	*In vitro In vivo* Clinical	Possible damage in the cell membrane	Albrecht et al., 2007; Conrad et al., 2013
*Arnica montana*	Not specified	Terpenoids	Uncharacterized sesquiterpene lactones	Biofilm modulating activity on UPEC	*In vitro*	None	Vacheva et al., 2011
*Avicennia marina*	Leaves	Phenolics	1,3-benzodioxole,5,5-(tetrahydro-1H,3H-furo[3,4-c]furan-1,4-diyl)bis-,[1s-(1 alpha,3a alpha,4 beta,6a alpha)]	Antibacterial activity	*In vitro*	None	Devi et al., 2014
*Betula pendula*	Leaves	Phenolics	3,40-dihydroxypropiophenone-3-β-d-glucoside, quercetin-3-galactoside, quercetin-3-glucuronide, caffeic acid derivatives, p-coumaric acid	Bactericidal activity	*In vitro*	Modifications in the bacterial surface structures responsible for binding to the occupied surface	Wojnicz et al., 2012
*Boerhaavia diffusa*	Hairy root, Root	None identified	None identified	Active against UPEC MDR strains	*In vitro*	None	Sahu et al., 2013
*Calluna vulgaris*	Leaves, flowers	Phenolics	Total phenols and flavonoids	Antibacterial activity	*In vitro*	None	Vucic et al., 2014
*Citrus reticulata*	Seeds	None identified	None identified	Reduction of UPEC invasion	*In vitro*	Decreased β1 integrin expression	Vollmerhausen et al., 2013
*Costus spicatus*	Leaves	Phenolics	Ferulic acid, caffeic acid, quercetin, apigenin.	Antimicrobial activity	*In vitro*	Correlation between the antioxidant and antimicrobial activity	Uliana et al., 2015
*Crateava nurvala*	Bark	None identified	None identified	Growth inhibition	*In vitro*	None	Chandra and Gupta, 2001
*Curcuma longa*	Rhizome	Phenolics	Curcumin	Antibiofilm activity, inhibition of swimming and swarming behavior, enhanced susceptibility of UPEC toward antibiotics	*In vitro*	Reduction in biofilm morphology and thickness	Packiavathy et al., 2014
*Cybopogum citratus*	Essential oils	Terpenoids	Myrcene, neral, geranial	Antimicrobial activity	*In vitro*	None	Pereira et al., 2004
*Cyperus rotundus*	Rhizome	Terpenoids	Saponins	Antibacterial activity	*In vitro*	None	Sharma et al., 2014
*Equisetum arvense*	Leaves	Phenolics	Quercetin dihexoside, kaempherol dihexoside, kaempherol-dirhamnosyl-hexoside, protocatechuic acid, caftaric acid, ferulic acid, caffeic acid	Antimicrobial activity, inhibition of biofilm mass production, antiadhesive	*In vitro*	Modifications in the bacterial surface structures responsible for binding to the occupied surface	Wojnicz et al., 2012; Rafsanjany et al., 2013
*Galium odoratum*	Leaves	Phenolics	Protocatechuic acid, caffeoylquinic isomer, quercetin and kaempherol derivatives, iridoids	Weak antimicrobial activity	*In vitro*	Modifications in the bacterial surface structures responsible for binding to the occupied surface	Wojnicz et al., 2012
*Gynostemma pentaphyllum*	Leaves	Terpenoids, dammarane-type saponins	Gypenosides	Reduction of pro-inflammatory response of BECs to UPEC; modulation of antimicrobial peptides	*In vivo*	NF-κB inhibition and ERK activation	Lüthje et al., 2015
*Herniaria glabra*	Leaves	Phenolics	Caffeoylquinic and feruloylquinic isomers, quercetin, kaempherol and isorhamnetin derivatives, iridoids,	High bactericidal activity, inhibition of biofilm mass production	*In vitro*	Modifications in the bacterial surface structures responsible for binding to the occupied surface	Wojnicz et al., 2012
*Labisia pumila var. alata*	Herbal	Phenolics	Gallic acid, anthocyanin	Induced uroepithelial apoptosis, reduces the number of intracellular UPEC in BECs	*In vitro*	Increased levels of caveolin-1, reduced expression of β1 integrin	Fazliana et al., 2011
*Lactuca indica*	Leaves	None identified	None identified	Decreases bacterial colonization of bladder epithelial cells, reduces phosphorylation of the focal adhesion kinase (FAK)	*In vitro*	Inhibition of FAK significantly decreases bacterial adherence	Lüthje et al., 2011
*Ocimum gratissimum*	Essential oils	Terpenoids	1,8 cineol, eugenol, methyl-eugenol, thymol, p-cimene, cis-ocimene, and cis-caryophyllene	Antimicrobial activity	*In vitro*	None	Pereira et al., 2004
*Orthosiphon stamineus*	Leaves	None identified	None identified	Antiadhesive effects	*In vivo*	Direct interaction of compounds from the extract with the bacterial adhesins	Rafsanjany et al., 2013
*Parkia biglobosa*	Root bark	None identified	Uncharacterized glycosides, tannins, saponins, alkaloids	Antibacterial activity	*In vitro*	None	El-Mahmood and Ameh, 2007
*Peganum. harmala*	Seeds	None identified	None identified	Antibacterial Activity	*In vitro*	None	Saeidi et al., 2015
*Petasites album*	Not specified	Terpenoids	Uncharacterized mono-, sesqui-, tri-terpenoids	Biofilm modulating activity on UPEC	*In vitro*	None	Vacheva et al., 2011
*Petasites hybridus*	Not specified	Terpenoids	Uncharacterized mono-, sesqui-, tri-terpenoids	Biofilm modulating activity on UPEC	*In vitro*	None	Vacheva et al., 2011
*Petroselinum crispum*	Leaves	Phenolics	Uncharacterized coumarins	Antibacterial activity	*In vitro*	None	Petrolini et al., 2013
*Piper arboreum*	Leaves	None identified	None identified	Modulatory activity, synergistic activity with antibiotic drugs	*In vitro*	None	Tintino et al., 2014
*Polygonum capitatum*	Whole plant	Terpenoids, phenolics	Tentative identification of gallic acid, quercitrin, catechin, flavonoids, triterpenoids, steroids	Anti-inflammatory, moderate antibacterial	*In vitro In vivo*	None	Liao et al., 2011
*Punica granatum*	Seed	None identified	None identified	Antibacterial activity	*In vitro*	None	Sharma et al., 2014
*Rhodiola rosea*	Not specified	Phenolics	Uncharacterized flavonoids, proanthocyanidins, phenylpropanoids	Biofilm modulating activity on UPEC	*In vitro*	None	Vacheva et al., 2011
*Rosmarinus officinalis*	Leaves	Phenolics	rosmarinic acid	Antibacterial activity	*In vitro*	None	Petrolini et al., 2013
*Salvia officinalis*	Essential oils	Terpenoids	1,8-cineole	Antimicrobial activity	*In vitro*	None	Pereira et al., 2004
*Salvia plebeia*	Whole plant	None identified	None identified	Diuretic activity, UPEC susceptibility	*In vivo*	None	Peng et al., 2010
*Schefflera leucantha*	Leaves	None identified	None identified	Antibacterial activity	*In vitro*	None	Sittiwet et al., 2009
*Toddalia asiatica*	Whole plant, leaves	Phenolics Alkaloids	Ulopetrol, Flindersine	Antibacterial Activity against MDR UPEC	*In vitro*	None	Raj et al., 2012; Venkatadri et al., 2015
*Tropaeoli majoris*	Leaves	Isothiocyanates	Benzyl-Isothiocyanate, Phenylethyl-Isothiocyanate	Intermediate susceptibility,	*In vitro In vivo* Clinical	Possible damage in the cell membrane	Albrecht et al., 2007; Conrad et al., 2013
*Urtica dioica*	Leaves	Phenolics	Protocatechuic, ferulic, p-coumaric, and dicaffeoylquinic acids,	Antimicrobial activity, antiadhesive effects	*In vitro In vivo*	Modifications in the bacterial surface structures responsible for binding to the occupied surface, direct interaction of compounds from the extract with the bacterial adhesins	Wojnicz et al., 2012; Rafsanjany et al., 2013
*Vaccinium vitis-idaea*	Leaves	Phenolics	Quercetin derivatives, derivatives of caffeoylquinic, caffeoyl-hexose-hydroxyphenol and coumaroyl-hexose-hydroxyphenol acids, procyanidins (A and B dimers), iridoids	High bactericidal activity, inhibition of biofilm mass production	*In vitro*	Modifications in the bacterial surface structures responsible for binding to the occupied surface	Wojnicz et al., 2012
*Vernonia amygdalina*	Leaves, stems	None identified	None identified	Antimicrobial activity	*In vitro*	None	Uzoigwe and Agwa, 2011
*Zea mays*	Stigmata	Phenolics	Derhamnosylmaysin, 3′-deoxyrhamnosylmaysin, 3′-O-methyl derhamnosylmaysin, apiferol, alternanthin	Decreased bacterial adhesion	*In vivo In vitro*	Interaction with bacterial outer membrane proteins	Rafsanjany et al., 2013, 2015
*Zingiber officinale*	Rhizome	None identified	None identified	Antibacterial activity	*In vitro*	None	Sharma et al., 2009

**Figure 11:**
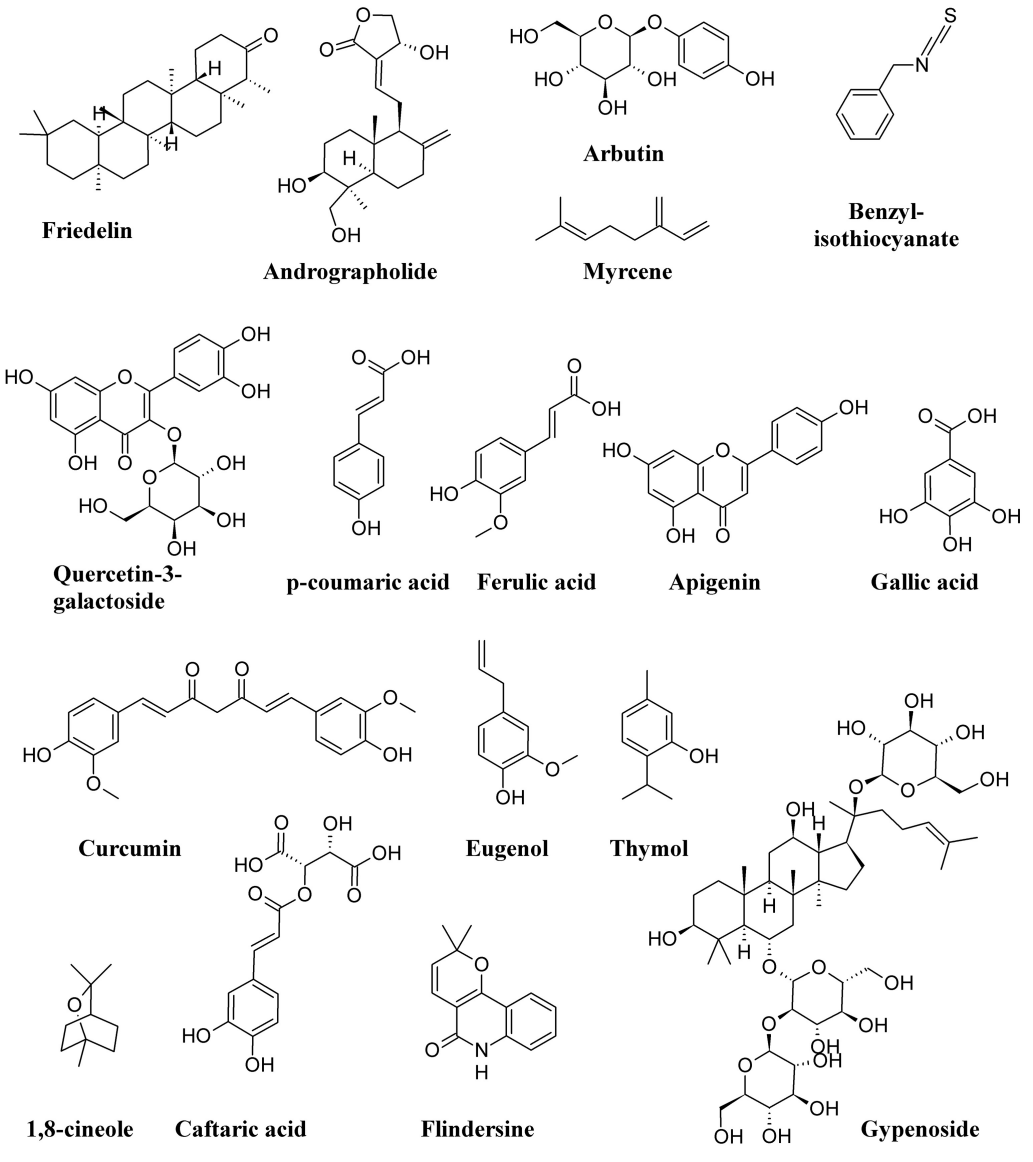
Structure formulae of some representative bioactive compounds isolated from plant extracts exerting antimicrobial activity against UPEC listed in Table 1.

Figure 11 shows the structure formulae of some bioactive compounds against UPEC listed in Table 1.

## Concluding remarks

Uropathogenic *Escherichia coli* infections pose a serious problem to human health, with societal costs of tens of billion US$ worldwide. The increased resistance to both synthetic and natural antibiotics causes recurrence and chronicity of infection, with the emergence of new and more serious illnesses. There are both direct and indirect strategies against UPEC infections. Direct strategies are targeting bacteria viability, bladder epithelium adhesion and biofilm formation; indirect strategies elicit and enhance immune responses, by stimulating infected tissues and cells to overreact to UPEC invasion.

**Figure 12:**
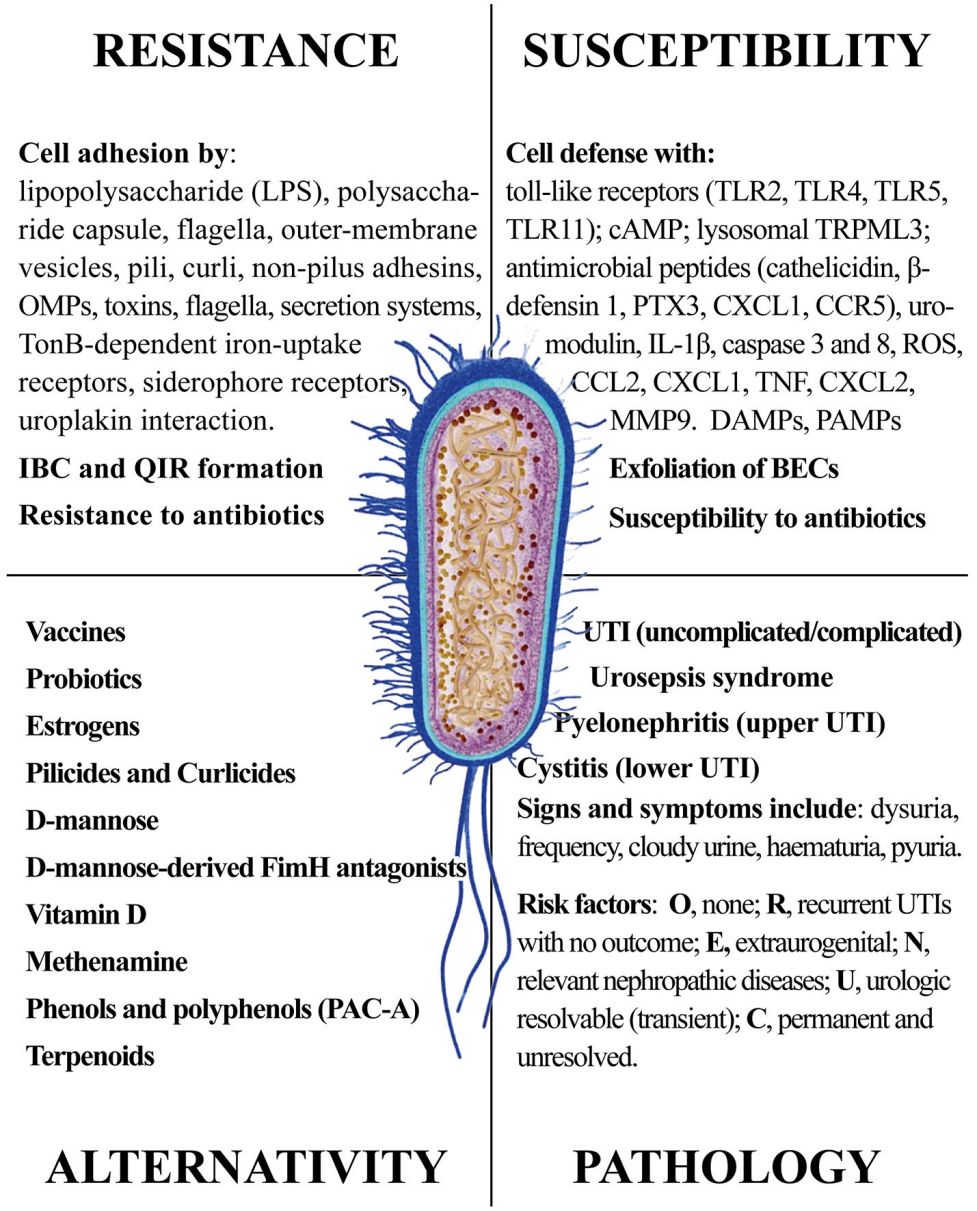
The four main areas representing the Strengths and the Weaknesses of UPEC, the Opportunities for alternative remedies to antibiotics and, finally, the Treats that UPEC cause to human health.

In this review, we provided a comprehensive picture of the UPEC infection, with the aim of giving the reader elements from both sides of the problem: the pathogen infectivity and the human cell response. The ongoing search for new and more effective antibiotics and the discovery of natural products from plants, fungi and non-pathogenic bacteria shall consider the multiple aspects of UPEC infection. Figure 12 summarizes the four main areas representing the Strengths and the Weaknesses of UPEC, the Opportunities for alternative remedies to antibiotics and, finally, the Treats that UPEC cause to human health. While antibiotics will remain the main drugs to reduce infections, alternative chemicals may offer the opportunity to (a) by-pass antimicrobial resistance and (b) show multi-targeted action, with the potential of targeting both the UPEC and the innate immune system. We cannot exclude that new therapies may include both antibiotics and natural products altogether, as recently demonstrated (Vadekeetil et al., 2016). An increasing body of evidence shows that reduction of adhesion of UPEC to urinary tract tissues reduces recurrence and increases recovery. Future interesting targets might not only be directed to UPEC adhesins, but also to immune-based strategies able to improve cell responses to UPEC infection; in this context, several natural products fit this strategy.

## Author contributions

MT, GG and MM wrote the manuscript.

### Conflict of interest statement

The authors declare that the research was conducted in the absence of any commercial or financial relationships that could be construed as a potential conflict of interest.
